# Citrate- and Succinate-Modified Carbonate Apatite Nanoparticles with Loaded Doxorubicin Exhibit Potent Anticancer Activity against Breast Cancer Cells

**DOI:** 10.3390/pharmaceutics10010032

**Published:** 2018-03-11

**Authors:** Sultana Mehbuba Hossain, Ezharul Hoque Chowdhury

**Affiliations:** Jeffrey Cheah School of Medicine and Health Sciences, Monash University Malaysia, Jalan Lagoon Selatan, Bandar Sunway, 47500 Petaling Jaya, Selangor, Malaysia; hossainmishu_1990@yahoo.com

**Keywords:** doxorubicin, carbonate apatite (CA), citrate, succinate, nanoparticles (NPs), cellular uptake, cytotoxicity, breast cancer

## Abstract

Biodegradable inorganic apatite-based particle complex is popular for its pH-sensitivity at the endosomal acidic environment to facilitate drug release following cellular uptake. Despite being a powerful anticancer drug, doxorubicin shows severe off-target effects and therefore would need a carrier for the highest effectiveness. We aimed to chemically modify carbonate apatite (CA) with Krebs cycle intermediates, such as citrate and succinate in order to control the growth of the resultant particles to more efficiently carry and transport the anticancer drug into the cancer cells. Citrate- or succinate-modified CA particles were synthesized with different concentrations of sodium citrate or sodium succinate, respectively, in the absence or presence of doxorubicin. The drug loading efficiency of the particles and their cellular uptake were observed by quantifying fluorescence intensity. The average diameter and surface charge of the particles were determined using Zetasizer. Cell viability was assessed by MTT assay. Citrate-modified carbonate apatite (CMCA) exhibited the highest (31.38%) binding affinity for doxorubicin and promoted rapid cellular uptake of the drug, leading to the half-maximal inhibitory concentration 1000 times less than that of the free drug in MCF-7 cells. Hence, CMCA nanoparticles with greater surface area enhance cytotoxicity in different breast cancer cells by enabling higher loading and more efficient cellular uptake of the drug.

## 1. Introduction

Breast cancer is the most common invasive cancer in women, causing more than 521,900 cancer deaths and 1,000,000 new cases per year all over the world [1]. Currently available chemotherapeutics are non-specific, exerting huge side effects on healthy cells [2,3]. Delivering cancer drugs specifically to a selected cancerous cell population to minimize the biodistribution of the drugs in healthy tissues, and achieving an optimum therapeutic effect at a lower drug concentration is one of the challenges in modern chemotherapy.

Doxorubicin (DOX) is a commonly used anticancer drug that inhibits macromolecular biosynthesis by interacting with the DNA. DOX produces its antineoplastic effect by intercalation of the planar anthracycline chromophore group between two base pairs of DNA. DOX inhibits topoisomerase II enzyme and stabilizes the topoisomerase II complex by relaxing supercoils in DNA; as a consequence, the DNA chain breaks down, preventing the replication process. Apart from this, DOX possesses the ability to generate free radicals and decrease mitochondrial oxidative phosphorylation, and thus induces DNA and cell membrane damage. However, the major toxic effect of DOX is cardiomyopathy, which may lead to congestive heart failure and death [4,5,6,7]. Besides its toxic effect, the inherent multidrug-resistant effect remains as a major problem to be solved.

Nanoparticles (NPs) are a highly promising and reliable approach in revolutionizing cancer therapy to improve drug accumulation in tumor sites to get a maximum therapeutic index with the decreased cytotoxicity in healthy tissues [8,9], by altering the pharmaceutical formulations and improving their pharmacokinetic properties [10]. Moreover, NPs are able to bypass p-glycoprotein-mediated drug resistance and take the advantage of the leaky vasculature and the poor lymphatic drainage system of tumor site to deliver anticancer drugs, known as the enhanced permeability and retention (EPR) effect [11].

Inorganic NPs are most popular for their physiochemical properties [12], such as the ease of formulation, controlled particle size and a very narrow size distribution. Inorganic NPs are advantageous over organic NPs in terms of site-specific drug delivery, stability and rapid release [13,14,15]. Liposomes, the most commonly used lipid nanoparticles, face some major difficulties including instability, poor batch-to-batch reproducibility, difficulties in sterilization and low drug loading. In contrast, polymeric nanoparticles especially fabricated by emulsion and related techniques are limited to the spherical shape, often plagued by polydispersity in size [16,17].

Inorganic pH-responsive carbonate apatite (CA) with a molecular formula of Ca_10_(PO_4_)_6-X_(CO_3_)_X_(OH)_2_, has emerged as an attractive non-viral carrier, as it is biodegradable, possesses heterogeneous surface charge, and inhibits crystal growth. CA NPs formed in a super-saturated solution of PO_4_^3−^, HCO_3_^−^ and Ca^2+^ ions provide a binding opportunity on a broader scale for both anionic and cationic drugs through electrostatic interactions, owing to the heterogeneous charge distribution on the particle surface. Drug-containing NPs can be internalized into the tumor cell by endocytosis, resulting in particle dissolution and release of the entrapped drug inside the endosomal acidic environment [18,19]. However, CA particles formed by exogenously adding Ca^2+^ tend to aggregate and form larger particles through ionic interactions [20], which can be stabilized by adding serum proteins [21]. Since citrate, a tricarboxylic acid of the Krebs’ cycle, is known to bind with free calcium and reduce platelet aggregation and fibrinogen binding [22,23], we hypothesized that it might electrostatically interact with Ca^2+^-rich domains of CA NPs during the fabrication process and thus limit their uncontrolled growth. Accordingly, citrate-modified CA (CMCA) NPs were prepared by adding sodium citrate along with exogenous Ca^2+^ to HCO_3_^−^-buffered media containing endogenous Ca^2+^ and phosphate. In order to explore the influence of the number of carboxylic acid groups in formation of nano-size particles and drug loading efficacy, sodium succinate, a dicarboxylic acid and an intermediate of Krebs’ cycle was also used to modify the particles, generating succinate-modified CA (SMCA) NPs [24].

## 2. Materials and Methods

### 2.1. Reagents

Dulbecco’s Modified Eagle Medium (DMEM) powder was purchased from Gibco by Life Technology (Thermo Fisher Scientific, Waltham, MA, USA). Calcium chloride dihydrate (CaCl_2_·2H_2_O), sodium bicarbonate (NaHCO_3_), sodium hydrogen phosphate (Na_2_HPO_4_), potassium dihydrogen phosphate (KH_2_PO_4_), sodium citrate tribasic dihydrate, sodium succinatedibasic hexahydrate and trypsin-ethylene diaminetetraacetate (EDTA) salts were obtained from Sigma-Aldrich (St. Louis, MO, USA). Dimethyl sulphoxide (DMSO), thiazolyl blue tetrazolium bromide (MTT), nonidet P40 substitute (NP40) and anti-cancer drug DOX were acquired from Sigma-Aldrich (St. Louis, MO, USA). DMEM liquid media, foetal bovine serum (FBS), TrypLE Express, and penicillin-streptomycin were procured from Sigma-Aldrich (St. Louis, MO, USA). Sodium chloride and potassium chloride salts were bought from Fischer Scientific (Loughborough, UK). Acetonitrile (ACN), hydrochloric acid (HCl), methanol and glycerol were from Fischer Scientific (Loughborough, UK). DOX was dissolved in distilled water and 8.62 mM stock solution was prepared.

### 2.2. Synthesis of CA, CMCA and SMCA NPs

#### 2.2.1. DOX-Loaded CA NPs

Apatite-based pH sensitive inorganic NPs were prepared by the precipitation method. Briefly, DMEM media was prepared by using the appropriate amount of DMEM powder and 44 mM sodium bicarbonate in Milli Q water and the final pH of the media was adjusted to 7.4 using 0.1 M hydrochloric acid [19,25]. In addition, 4 mM exogenous calcium was taken with 0 µM to 20 µM of DOX in Eppendorf tubes and added to 1 mL of serum-free freshly prepared DMEM, followed by incubation at 37 °C for 30 min to generate DOX-loaded CA NPs. DOX at 0 µM, 5 µM, 10 µM, 15 µM and 20 µM concentration was added to 1 mL fresh serum-free DMEM solution, which was then incubated at 37 °C for 30 min. The turbidity of free DOX and Dox-loaded CA NPs was measured at 320 nm wavelength by UV-VIS spectrophotometer (Jasco, Oklahoma City, OK, USA).

#### 2.2.2. DOX-Loaded CMCA NPs

CMCA NPs were synthesized by adding 1 mM of sodium citrate tribasic dihydrate along with 4 mM of exogenous Ca^2+^ to 1 mL fresh serum-free DMEM solution, followed by incubation at 37 °C for 30 min. DOX at 0 to 20 µM was added with 4 mM exogenous calcium and 1 mM sodium citrate to 1 mL of freshly prepared DMEM solution to generate DOX-loaded CMCA NPs through incubation for 30 min at 37 °C. The turbidity of generated particle suspension was measured at 320 nm wavelength by UV-VIS spectrophotometer.

#### 2.2.3. Succinate-Modified CA (SMCA) NPs

Sodium succinate dibasic hexahydrate at 1 mM to 16 mM, and exogenous Ca^2+^ at 4 mM were added to 1 mL of serum-free freshly prepared DMEM solution. The mixture was then incubated for 30 min at 37 °C to form succinate-modified CA (SMCA) NPs. The turbidity of the particle suspension was measured according to the above-mentioned protocol by UV-VIS spectrophotometer.

#### 2.2.4. DOX-Loaded SMCA NPs

DOX was added at 0 µM to 20 µM concentration, along with 4 mM exogenous calcium and 4 mM sodium succinate to 1 mL of freshly prepared DMEM solution to generate DOX-loaded SMCA NPs through incubation for 30 min at 37 °C. The turbidity of generated particle suspension was measured according to the above-mentioned protocol by UV-VIS spectrophotometer.

### 2.3. Optical Images of Free DOX, DOX-Loaded CA and DOX-Loaded CMCA and SMCA Particles

MCF-7 cell lines were treated with 1 µM and 5 µM of free drug, drug-loaded CA and CMCA nanoparticles. Free DOX and DOX-loaded apatite NPs with 10% FBS were used to treat the cells for 1 h, and 4 h in the incubator for cellular internalization. Extracellular particles were removed by 5 mM EDTA and subjected to PBS washing prior to observation of the cells. The images of internalized particles were taken by Olympus Fluorescence Microscope IX81 (Shinjuku, Tokyo, Japan) with 4× and 20× magnifications at a scale bar of 100 µm and 20 µm, respectively.

### 2.4. Estimation of Drug Binding Affinity towards NPs

DOX was dissolved at 0 µM, 1 µM, 5 µM, 10 µM, 15 µM and 20 µM concentrations in 1mL PBS and quantified with an excitation wavelength of 485 nm and an emission wavelength of 535 nm using PerkinElmer 2030 manager software (CellSens Dimension) attached with 2030 multilabel reader victor X5 (PerkinElmer, Waltham, MA, USA). The standard curve was made by plotting concentrations vs. fluorescence intensity. DOX at 5 µM or 10 µM and exogenous calcium at 4 mM were added to 1 mL of freshly prepared DMEM to form DOX-loaded CA NPs. Similarly, DOX was added at 5 µM or 10 µM along with 4 mM exogenous calcium and 1 mM sodium citrate to 1 mL of freshly prepared DMEM solution to formulate DOX-loaded CMCA NPs. DOX-incorporated SMCA NPs were prepared by adding 5 µM or 10 µM of DOX, and 4 mM of exogenous calcium along with 4 mM sodium succinate to 1 mL of freshly prepared DMEM media. The mixture was then incubated at 37 °C for 30 min. Afterwards, the particle suspension was centrifuged at 6000 rpm for 20 min at 4 °C with a refrigerated bench-top microcentrifuge. The supernatant was discarded and 1 mL fresh DMEM media was added to the pellet prior to centrifugation at 6000 rpm for 20 min. Finally, after removing supernatant, the pellet was mixed with 10 mM EDTA in PBS to dissolve the particles and release the bound drugs, and subjected to fluorescence intensity measurement.

The concentrations of DOX present in the suspension were calculated from the fluorescence intensity, using the standard curve. Data were represented as % of binding affinity (or % loading efficiency) of DOX to NPs. The % binding efficiency was calculated using the following formula:

(1) $$\%\;\mathrm{DOX}\;\mathrm{release}\;\mathrm{form}\;\mathrm{NPs}=\frac{\left[X\right]\mathrm{NP}\;\mathrm{bound}\;\mathrm{drug}\;\mathrm{at}\;\mathrm{pH}\;7.5-\;\left[Y\right]\mathrm{NP}\;\mathrm{bound}\;\mathrm{drug}\;\mathrm{at}\;\mathrm{different}\;\mathrm{pH}}{\left[Y\right]\mathrm{NP}\;\mathrm{bound}\;\mathrm{drug}\;\mathrm{at}\;\mathrm{pH}\;7.5}$$

where “[Y] NP bound drug” is the fluorescence intensity of DOX bound with NPs and “[Y] initial” is the fluorescence intensity of DOX used to prepare standard curve by using a flurospectrophotometer. DOX bound to NPs was calculated by using the standard curve. Each of the experiments was done in triplicate and presented as mean ± standard deviation (SD).

### 2.5. Particle Size and Zeta Potential Measurement

DOX was added at 100 pM, 1 nM, 10 nM, 100 nM and 1 µM concentrations, along with 4 mM exogenous Ca^2+^ and 1 mM sodium citrate to 1 mL of freshly prepared DMEM in order to generate DOX-loaded CMCA NPs. DOX in the same concentrations was added with 4 mM exogenous calcium to 1 mL of freshly prepared DMEM solution to generate DOX-loaded CA NPs. Sodium succinate at 1 mM to 16 mM, and exogenous Ca^2+^ at 4 mM were added to 1 mL of freshly prepared DMEM solution to prepare drug-free SMCA particles. The suspension was incubated at 37 °C temperature for 30 min, followed by addition of 10% FBS of the total volume. All the formulations were preserved in the ice chiller during measurement. Particle size and surface charge measurement were done by Malvern nano zetasizer (Malvern, Worcestershire, UK), with attached zetasizer software version 6.20 for data analysis.

### 2.6. Cell Culture and Seeding

MCF-7 cells, which is a human breast cancer cell line and 4T1 cells, which is a mouse breast cancer cell line were used in this study. All cell lines were cultured in separate 25 cm^2^ flask at pH 7.4 with complete DMEM media containing 10% FBS, 1% penicillin and streptomycin antibiotic. The cells were cultured in a humidified atmosphere containing 5% CO_2_ at 37 °C. Both cell lines from the exponential growth phase were trypsinized, washed through repeated centrifugation steps and seeded at 50,000 cells per well into 24-well plates (Greiner, Frickenhause, Germany) the day before treatments with only drug and drug-loaded NPs.

### 2.7. Cell Treatment with Free DOX and DOX-Apatite Complexes

Fresh DMEM solution was prepared according to above-mentioned protocol, adjusting the pH to 7.4 using 0.1 M hydrochloric acid. DOX was added at 1 pM, 10 pM, 100 pM, 1 nM, 10 nM, 100 nM and 1 µM, along with 4 mM exogenous calcium and 1 mM sodium citrate to 1 mL of filtered DMEM solution. This mixture was then incubated for 30 min at 37 °C to generate DOX-loaded CMCA NPs. Similarly, the same concentrations of DOX were added with 4 mM exogenous calcium and 4 mM sodium succinate to 1 mL of filtered DMEM solution to generate DOX-loaded SMCA NPs. The same concentrations of DOX were added to 1 mL DMEM solution prior to incubation in the same manner to prepare NP-free drug solution. DOX was added at the same concentrations with 4 mM exogenous calcium to 1 mL of filtered DMEM media to generate DOX-loaded CA NPs. Drug-free SMCA NPs were also fabricated by the addition of sodium succinate at 1 mM to 16 mM concentrations. To avoid further particle formation during cell treatment, 10% FBS was added to drug-free NPs and DOX-loaded NPs. The complete DMEM medium in each well was substituted with free DOX, particles alone or DOX-loaded particles. Afterwards, the 24-well plates were placed in the incubator for two consecutive days until the cell viability was assessed.

### 2.8. MTT Assay in MCF-7 and 4T1 Cell Lines

After 48 h of treatment, 50 μL of MTT (5 mg/mL in PBS) was added to each well of the plates to form formazan crystals by metabolically active cells, which were subsequently incubated in the incubator humidified with CO_2_ (5%) for 4 h at 37 °C. Later, 300 μL of DMSO solution was added following removal of the MTT medium. The plates were then agitated on microplate reader for 10 min to dissolve the purple formazan crystals properly. The absorbance was measured by spectrophotometric microplate reader (BIO-RAD-Microplate Reader, Hercules, CA, USA) at a wavelength of 595 nm with a reference wavelength to 630 nm. Each experiment was done in triplicate with the data plotted in graphs as mean ± SD of three independent experiments.

### 2.9. Data Analysis

The cell viability was calculated by using the absorbance values achieved by MTT assay using the following formula:

(2) $$\%\;\mathrm{Cell}\;\mathrm{viability}=\frac{\mathrm{Absorbance}\;\mathrm{of}\;\mathrm{treated}\;\mathrm{sample}}{\mathrm{Absorbance}\;\mathrm{of}\;\mathrm{control}}\;\times 100\%$$

The cytotoxicity (%) of the free drugs and drug-loaded NPs were measured by the following formula:

(3) $$\%\;\mathrm{cytotoxicity}={\mathrm{CV}}_{\mathrm{free}\;\mathrm{drug}}\;–\;{\mathrm{CVNP}}_{\mathrm{NP}\;\mathrm{drug}}.$$

CV_free_ _drug_ is the cell viability (%) of free drug and CV_NP drug_ is the cell viability (%) of NP bound drugs.

The enhancement of cytotoxicity (%) of the drugs loaded into NPs was measured using the following formula:

(4) $$\%\;\mathrm{Cytotoxicity}\;\mathrm{of}\;\mathrm{NPs},\;x=100-\left(\frac{\mathrm{Absorbance}\;\mathrm{of}\;\mathrm{treated}\;\mathrm{sample}\;\mathrm{with}\;\mathrm{NPs}}{\mathrm{Absorbance}\;\mathrm{of}\;\mathrm{control}}\times 100\%\right)$$

(5) $$\%\;\mathrm{Cytotoxicity}\;\mathrm{of}\;\mathrm{free}\;\mathrm{DOX},\;y=\;100-\left(\frac{\mathrm{Absorbance}\;\mathrm{of}\;\mathrm{treated}\;\mathrm{sample}\;\mathrm{with}\;\mathrm{only}\;\mathrm{drug}}{\mathrm{Absorbance}\;\mathrm{of}\;\mathrm{control}}\;\times 100\%\right)$$

(6) % Enhancement of Cytotoxicity=(m−n)−(o+p)

*m* = cell viability of the untreated cells, *o* = % cytotoxicity of NPs, *p* = % cytotoxicity of free drugs and *n* = % cell viability of DOX-loaded apatite-based NPs. Each experiment was performed in triplicate and shown as mean ± SD.

### 2.10. Cellular Uptake of Free DOX and DOX-Loaded NPs in MCF-7 Cell Lines

Being fluorescent in nature, DOX was quantified fluorometrically to measure cellular uptake of the drug in free and NPs-bound forms. The cellular uptake efficiency of free DOX, DOX-loaded CA and DOX-loaded CMCA prepared by using 5 µM or 10 µM concentration of DOX was investigated at 1 h and 4 h of treatment. MCF-7 cells were seeded into 24-well plates with a density of 50,000 cells per well and incubated at 37 °C overnight. Each well was treated with free DOX and DOX-loaded NPs with 10% FBS. The supernatant of culture media was discarded at 1 h and 4 h of the treatment. For qualitative analysis of drug uptake, the cells were treated briefly with 10 mM EDTA in PBS followed by washing with complete media and observation under a fluorescence microscope, whereas, for the quantitative analysis, the cells were washed three times with PBS and lysed with a lysis buffer and 200 µL lysate was withdrawn to determine the exact amount of drug internalized by the cells using a flurospectrophotometer. Fluorescence intensity of the treated cells was measured with an excitation at 485 nm and an emission at 535 nm using PerkinElmer 2030 manager software attached with 2030 multilabel reader victor X5 (PerkinElmer, Waltham, MA, USA).

Finally, the cellular uptake was calculated by using standard curve equation and the following formula:

(7) $$\%\;\mathrm{Cellular}\;\mathrm{uptake}=\frac{\mathrm{Internalized}\;\mathrm{drug}\;\mathrm{fluorescence}\;\mathrm{intensity}\;}{\mathrm{Initial}\;\mathrm{drug}\;\mathrm{fluorescence}\;\mathrm{intensity}}\;\times 100$$

Each experiment was done in triplicate and presented as mean ± SD.

### 2.11. Estimation of Bound Citrate in CMCA NPs

Sodium citrate of 0 mM, 5 mM, 10 mM, 15 mM, 20 mM, 25 mM and 30 mM concentrations was dissolved in distilled water and quantified by high-performance liquid chromatography (HPLC, Agilent, Santa Clara, CA, USA) to make the standard curve by plotting concentrations vs. peak areas. Sodium citrate was added at 8 mM or 16 mM concentration, along with 4 mM exogenous calcium to 1 mL of freshly prepared DMEM solution to formulate CMCA NPs. The mixture was then incubated at 37 °C for 30 min. Afterwards, the particle suspension was centrifuged at 6000 rpm for 20 min at 4 °C with a refrigerated bench-top microcentrifuge. The supernatant was collected and the amount of citrate present in the supernatant was analyzed by HPLC (Agilent, Santa Clara, CA, USA) attached with Agilent chemostation software, using zorbax C18 column (4.6 × 150 mm, 5 μm, Agilent). The mobile phase A was HPLC-grade filtered water while the mobile phase B was HPLC-grade 100% methanol, with the solvent being distilled water. The mobile phase A and B were used in 25:75 ratio. The flow rate, injection volume, detection wavelength (DAD) and stop time were set to 1.0 mL/min, 20 µL, 250 nm and 10 min, respectively. The concentrations of citrate present in the supernatant were calculated from the peak area, using the standard curve. Each of the experiments was done in triplicate and presented as mean ± standard deviation (SD).

### 2.12. pH Sensitivity of CA, CMCA and SMCA NPs

CA NPs were formulated using 20 mM Ca^2+^, CMCA NPs were prepared with 20 mM Ca^2+^ and 5 mM sodium citrate and SMCA NPs were synthesized using 20 mM Ca^2+^ and 20 mM sodium succinate in 200 µL PCR tubes. Each of the reaction mixtures was incubated at 37 °C for 30 min to generate the NPs which were subsequently suspended into 800 µL DMEM media of different pHs (7.5, 7.0, 6.5, 6.0, 5.5 and 5.0), prior to measurement of absorbance at 320 nm wavelength by UV-VIS spectrophotometer. Data were represented as mean ± SD in triplicate.

### 2.13. Release Profile of DOX

DOX-loaded CA NPs were formulated using 5 µM DOX and 20 mM Ca^2+^, DOX-loaded CMCA NPs were prepared with 5 µM DOX, 20 mM Ca^2+^ and 5 mM sodium citrate and DOX-loaded SMCA NPs were synthesized using 5 µM DOX, 20 mM Ca^2+^ and 20 mM sodium succinate in 200 µL PCR tubes. Each of the reaction mixtures was incubated at 37 °C for 30 min to generate the NPs, which were subsequently suspended into 800 µL DMEM media of different pHs (7.5, 7.0, 6.5, 6.0, 5.5 and 5.0). Afterwards, the particle suspensions were centrifuged at 6000 rpm for 20 min at 4 °C with a refrigerated bench-top microcentrifuge. The supernatant was discarded and the pellet was mixed with 10 mM EDTA in PBS to dissolve the particles and release the bound drugs, prior to fluorescence intensity measurement with an excitation wavelength of 485 nm and an emission wavelength of 535 nm using PerkinElmer 2030 manager software attached with 2030 multilabel reader victor X5 (PerkinElmer, Waltham, MA, USA).

The concentrations of DOX present in the suspension were calculated from the fluorescence intensity, using the standard curve. Data were represented as % of DOX release from NPs. The % DOX release profile was calculated using the following formula:

(8) $$\%\;\mathrm{DOX}\;\mathrm{release}\;\mathrm{form}\;\mathrm{NPs}=\frac{\left[X\right]\mathrm{NP}\;\mathrm{bound}\;\mathrm{drug}\;\mathrm{at}\;\mathrm{pH}\;7.5-\;\left[Y\right]\mathrm{NP}\;\mathrm{bound}\;\mathrm{drug}\;\mathrm{at}\;\mathrm{different}\;\mathrm{pH}\;}{\left[Y\right]\mathrm{NP}\;\mathrm{bound}\;\mathrm{drug}\;\mathrm{at}\;\mathrm{pH}\;7.5}\;\times 100\%$$

where “[Y] NP bound drug at different pH” is the fluorescence intensity of DOX bound with NPs at pH 7.0, 6.5, 6.0, 5.5 and 5.0 and “[Y] NP bound drug at pH 7.5” is the fluorescence intensity of DOX bound with NPs at pH 7.5. Data were represented as mean ± SD for triplicates.

### 2.14. Characterization of NPs by Fourier Transform-Infrared Spectroscopy (FTIR)

According to the above-mentioned protocol, CA NPs were prepared by using 40 mM Ca^2+^ and SMCA NPs were formulated with the same Ca^2+^ concentration along with sodium succinate, with Ca^2+^ to succinate ratio of 1:1. CMCA NPs was formulated similarly with Ca^2+^ (40 mM) to citrate (10 mM) ratio of 4:1. The fabricated NPs were centrifuged at 4000 rpm for 20 min and the precipitated pellets were lypholized by using a freeze dryer (Labconco, Kansas City, MO, USA). The spectrum of only sodium succinate salt, CA NPs, SMCA NPs and CMCA NPs were checked by Varian FTIR using the Varian Resolution Pro 640 software (Agilent, Santa Clara, CA, USA).

### 2.15. Characterization of NPs by Field Emission Scanning Electron Microscope (FE-SEM)

CA, CMCA and SMCA NPs were centrifuged at 13,000 rpm for 15 min with a refrigerated bench-top microcentrifuge. The supernatant was removed and 1 mL Milli-Q water was added to the pellet prior to centrifugation at 13,000 rpm for 15 min. Finally, after removing supernatant, the pellet was resuspended in 30 µL Milli-Q water. In addition, 5 μL of each sample was withdrawn and placed onto carbon tape-coated sample holder and dried at room temperature, followed by platinum sputtering of the dried samples with 30 mA sputter current at 2.30 tooling factor for 40 s. The size and morphology of sputtered NPs were visualized at 5 kV using FE-SEM (Hitachi/SU8010, Tokyo, Japan).

### 2.16. Characterization of NPs by Dynamic Light Scattering (DLS)

CA particles were formed using 4 mM exogenously added Ca^2+^ in 1 mL of freshly prepared DMEM. 1 mM sodium citrate and 4 mM exogenous Ca^2+^ were added to 1 mL of freshly prepared DMEM solution to prepare CMCA particles. Similarly, sodium succinate at 4 mM and exogenous Ca^2+^ at 4 mM were added to 1 mL of freshly prepared DMEM solution to prepare SMCA particles. The suspension was incubated at 37 °C for 30 min, followed by addition of 10% non-filtered FBS. All the formulations were preserved in the 4 °C ice chiller during measurement. DLS of NPs was measured in 5.50 mm position at a refractive index (RI) of 1.330 and viscosity 0.8872 at 25 °C temperature using a Malvern nano zetasizer, with attached zetasizer software for data analysis.

### 2.17. Statistical Analysis

Statistical analysis was performed using SPSS version 23 (Armonk, NY, USA) for Windows. All values were analyzed with one-way ANOVA. The results were considered as statistically significant at *p* < 0.05 with 95% confidence interval (CI). All the results have been presented as a mean ± SD.

## 3. Results

### 3.1. Synthesis and Characterization of DOX-Loaded CA, CMCA and SMCA NPs

**Figure 1:**
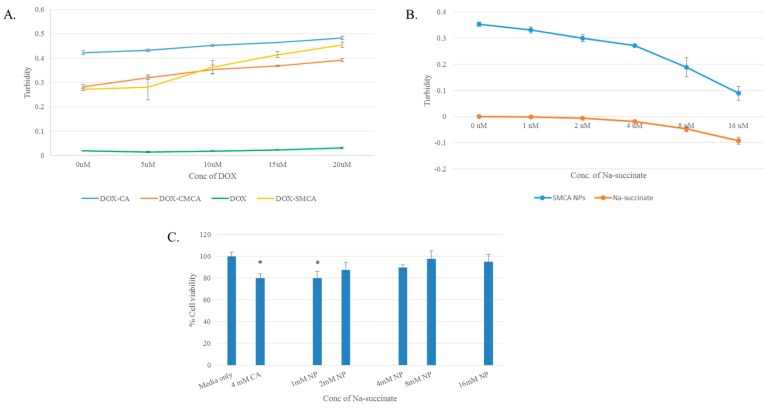
(**A**) Turbidity of DOX, DOX-loaded CA, DOX-loaded CMCA and DOX-loaded SMCA NPs measured using different concentrations (0 µM to 20 µM) of DOX; (**B**) turbidity of SMCA NPs measured using different concentrations (0 mM to 16 mM) of sodium succinate; (**C**) cell viability assessment by MTT assay after 48 h incubation of the CMCA NPs with MCF-7 cell line. Values were significant (*) at *p*-value 0.01 to 0.05 vs. untreated cell at CI of 95%. DOX: Doxorubicin, CA: Carbonate apatite, CMCA: Citrate-modified carbonate apatite, SMCA: Succinate-modified carbonate apatite, MTT: 3-(4,5-Dimethylthiazol-2-yl)-2,5-Diphenyltetrazolium Bromide and CI = Confidence interval.

The generation of DOX-loaded CA, CMCA and SMCA NPs, and empty NPs were characterized by UV-VIS spectrophotometer and optical image analysis. The UV-VIS spectra of DOX-loaded CA NPs and DOX-loaded CMCA NPs showed a gradual increase in absorbance (i.e., turbidity) at 320 nm wavelength with increasing concentration of DOX, whereas the same concentrations of the drug without NPs demonstrated no changes in the absorption spectra, suggesting that DOX might bind to the apatite-based particles and accelerate the growth of particle formation (Figure 1A). Figure 1B revealed the effect of sodium succinate on the particle formation using 4 mM of Ca^2+^. The turbidity of SMCA NPs was sharply decreased with increasing concentration of sodium succinate probably due to its growth inhibitory effect through interactions with Ca^2^-rich domains of NPs, as indicated by optical image analysis (data not shown). A large number of aggregated particles were observed for CA NPs formed with 4 mM Ca^2+^; however, the number and size of such aggregated particles were reduced with increasing doses of succinate (1–16 mM).

### 3.2. Particle Size and Surface Charge

**Figure 2:**
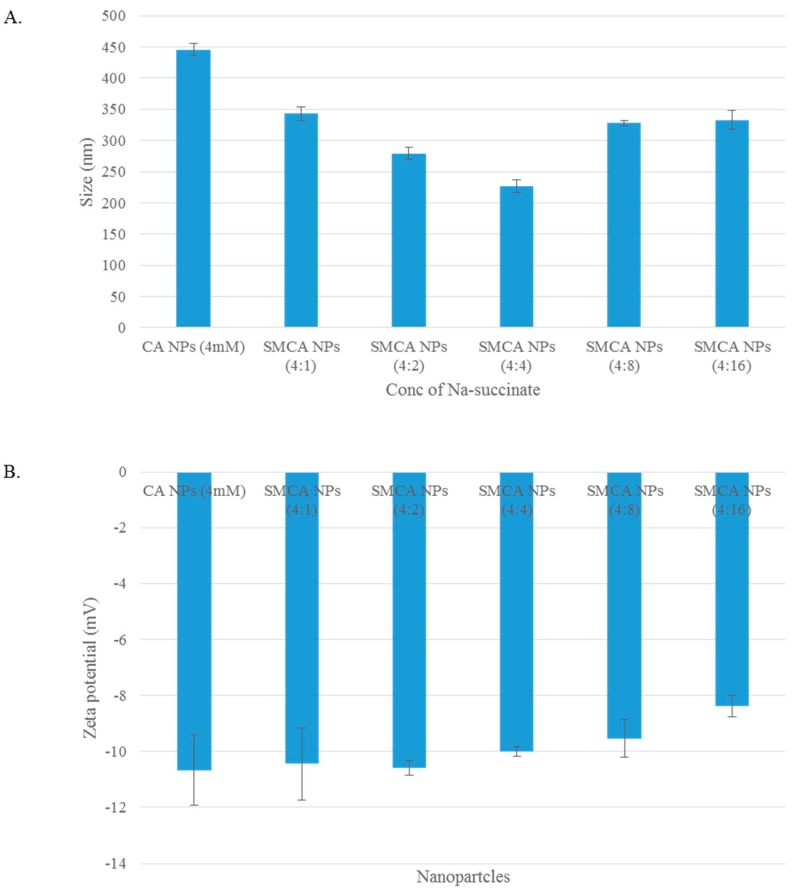
(**A**) Size of SMCA NPs; (**B**) zeta potential of SMCA NPs.

The cellular uptake through endocytosis depends on the size, shape and surface charge of NPs [25]. Hence, the size and zeta potential of CA, CMCA and SMCA NPs were measured. The average size of CA NPs formed with 4 mM Ca^2+^ was around 446 nm, while the average size of CMCA NPs formulated with 1 mM concentration of sodium citrate was approximately 107 nm (data not shown). SMCA NPs revealed dramatic behaviour in terms of particle size. Sodium succinate added at 1 mM, 2 mM and 4 mM concentrations along with 4 mM Ca^2+^ reduced the particle size of SMCA NPs to 343 nm, 279.5 nm and 226.5 nm, respectively (Figure 2A). However, the particle size was increased with a further increase in sodium succinate level, which might be due to its potential ability of cross-linking between particles at the higher concentration. At 8 mM and 16 mM concentrations of sodium succinate, the average particle sizes of SMCA NPs were 328 nm and 333 nm, respectively.

**Figure 3:**
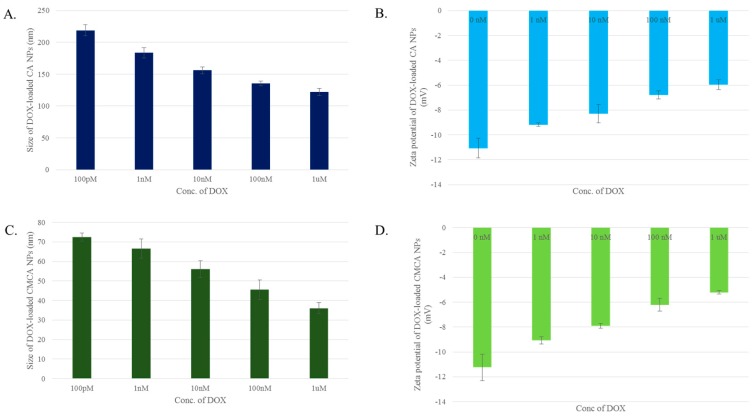
Size and zeta potential of DOX-loaded CMCA and CA NPs. (**A**) size of DOX-loaded CA NPs; (**B**) zeta potential of DOX-loaded CA NPs; (**C**) size of DOX-loaded CMCA NPs; (**D**) zeta potential of DOX-loaded CMCA NPs.

The size of DOX-loaded CA NPs and CMCA NPs were measured with the results indicating that DOX dose could control the particle size. The average diameters of DOX-loaded CA NPs were 219 nm, 183.5 nm, 156 nm, 135.5 nm and 122 nm when 100 pM, 1 nM, 10 nM, 100 nM and 1 µM concentrations of DOX were used, respectively, along with 4 mM Ca^2+^ (Figure 3A). The similar trend of drug concentration-dependent particle size reduction was noted with DOX-loaded CMCA NPs (Figure 3C). At 100 pM, 1 nM, 10 nM, 100 nM and 1 µM concentrations of DOX, the particle sizes of DOX-incorporated CMCA NPs were 72.5 nm, 66.5 nm, 56 nm, 45.5 nm and 36 nm, respectively. 

SMCA NPs had no apparent change in the zeta potential of the particles. The zeta potential remained in the range of −8.31 to −10 mV for all the SMCA NPs (Figure 2B). The zeta potential of DOX-incorporated CA and CMCA NPs were more electropositive than the free NPs. The zeta potential of CA NPs and CMCA NPs was almost −11 mV (data not shown), whereas the highly concentrated DOX (1 µM) incorporation reduced the electronegativity to almost −5.22 mV, suggesting that DOX-apatite particle formation is based on electrostatic interactions (Figure 3B,D). The positively charged DOX might bind electrostatically with negatively charged domains (rich in bicarbonate, phosphate or citrate) on apatite-based NPs, causing the net charge of the DOX-loaded NPs more electropositive than that of the free NPs.

### 3.3. Estimation of DOX Adsorption onto Apatite-Based NPs

**Figure 4:**
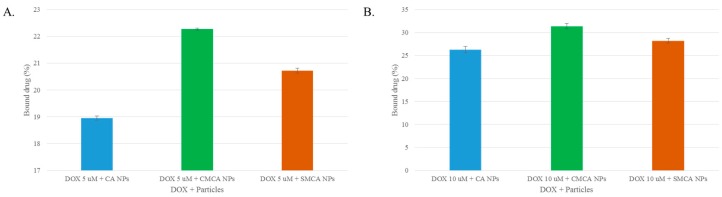
Fluorescence intensity analysis of free DOX and DOX-loaded CA and CMCA NPs. (**A**) drug binding efficiency (%) of CA, CMCA and SMCA NPs for 5 µM DOX; (**B**) drug binding efficiency (%) of CA, CMCA and SMCA NPs for 10 µM DOX.

Improving efficiency of drug binding to NPs and delivering it into the tumor cells are two prerequisites for ensuring therapeutic efficacy. The drug loading efficiency varies depending on drug delivery systems [26]. At 5 µM concentration, DOX possessed 18.95%, 20.72% and 22.27% binding affinity for CA, SMCA and CMCA NPs, respectively (Figure 4A). DOX showed a concentration-dependent binding affinity towards CA, SMCA and CMCA NPs. Thus, at 10 µM concentration, DOX presented almost 26.25%, 28.20% and 31.38% binding affinity for CA, SMCA and CMCA NPs, respectively (Figure 4B).

### 3.4. Cytotoxicity of Free DOX and DOX-Loaded CA, CMCA and SMCA NPs

The cytotoxicity of NPs against MCF-7 cells and 4T1 cells was assessed by using MTT assay. The cytotoxicity of SMCA NPs formulated with different concentrations (1–16 mM) of sodium succinate was measured, showing no apparent decrease in cell viability in comparison to CA NPs, indicating that SMCA NPs are as safe as CA NPs, providing no additional cytotoxicity (Figure 1C).

**Figure 5:**
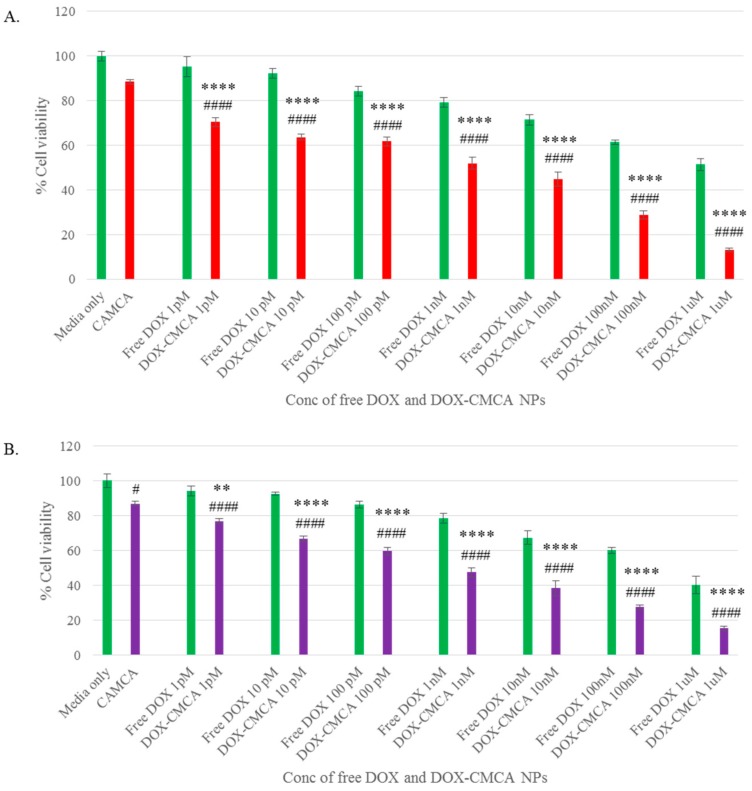
Cytotoxicity assay of DOX-loaded CMCA NPs. (**A**) cell viability assessment by MTT assay after 48 h incubation of the free DOX and DOX-loaded CMCA NPs with MCF-7 cell line; (**B**) cell viability assessment by MTT assay after 48 h incubation of the free DOX and DOX-loaded CMCA NPs with 4T1 cell line. Values were significant (*) at *p*-value 0.01 to 0.05, very significant (**) at *p*-value 0.001 to 0.01, highly significant (***) at *p*-value 0.0001 to 0.001, extremely significant (****) at *p*-value < 0.0001 vs. same treatment of free DOX. Values were significant (#) at *p*-value 0.01 to 0.05, very significant (##) at *p*-value 0.001 to 0.01, highly significant (###) at *p*-value 0.0001 to 0.001, extremely significant (####) at *p*-value < 0.0001 vs. untreated cells at CI of 95%.

**Figure 6:**
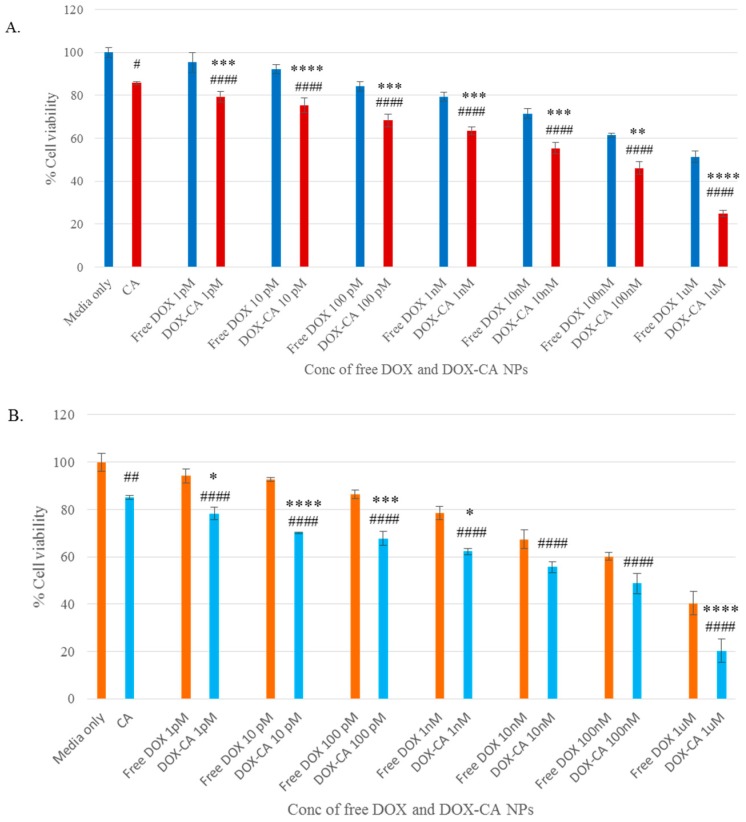
Cytotoxicity assay of DOX-loaded CA NPs. (**A**) cell viability assessment by MTT assay after 48 h incubation of the free DOX and DOX-loaded CA NPs with MCF-7 cell line; (**B**) cell viability assessment by MTT assay after 48 h incubation of the free DOX and DOX-loaded CA NPs with 4T1 cell line. Values were significant (*) at *p*-value 0.01 to 0.05, very significant (**) at *p*-value 0.001 to 0.01, highly significant (***) at *p*-value 0.0001 to 0.001, extremely significant (****) at *p*-value < 0.0001 vs. same treatment of free DOX. Values were significant (#) at *p*-value 0.01 to 0.05, very significant (##) at *p*-value 0.001 to 0.01, highly significant (###) at *p*-value 0.0001 to 0.001, extremely significant (####) at *p*-value < 0.0001 vs. untreated cells at CI of 95%.

**Figure 7:**
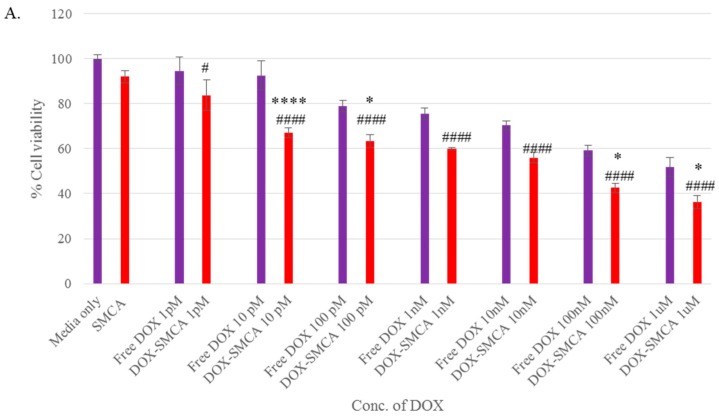

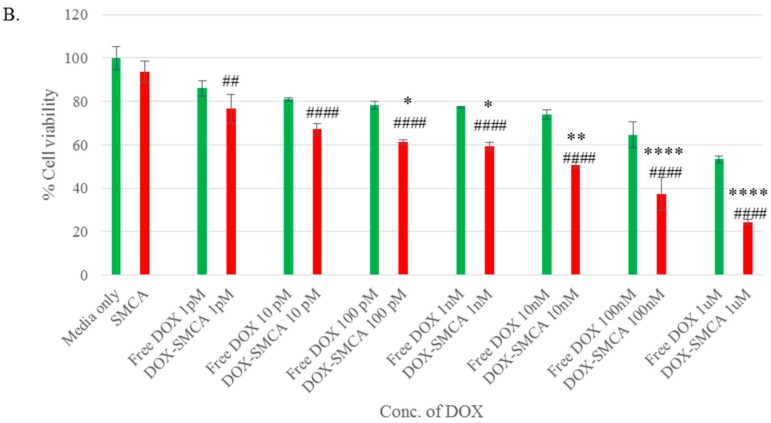
Cytotoxicity assay of DOX-loaded SMCA NPs. (**A**) cell viability assessment by MTT assay after 48 h incubation of the free DOX and DOX-loaded SMCA NPs with MCF-7 cell line; (**B**) cell viability assessment by MTT assay after 48 h incubation of the free DOX and DOX-loaded SMCA NPs with 4T1 cell line. Values were significant (*) at *p*-value 0.01 to 0.05, very significant (**) at *p*-value 0.001 to 0.01, highly significant (***) at *p*-value 0.0001 to 0.001, extremely significant (****) at *p*-value < 0.0001 vs. same treatment of free DOX. Values were significant (#) at *p*-value 0.01 to 0.05, very significant (##) at *p*-value 0.001 to 0.01, highly significant (###) at *p*-value 0.0001 to 0.001, extremely significant (####) at *p*-value < 0.0001 vs. untreated cells at CI of 95%.

**Figure 8:**
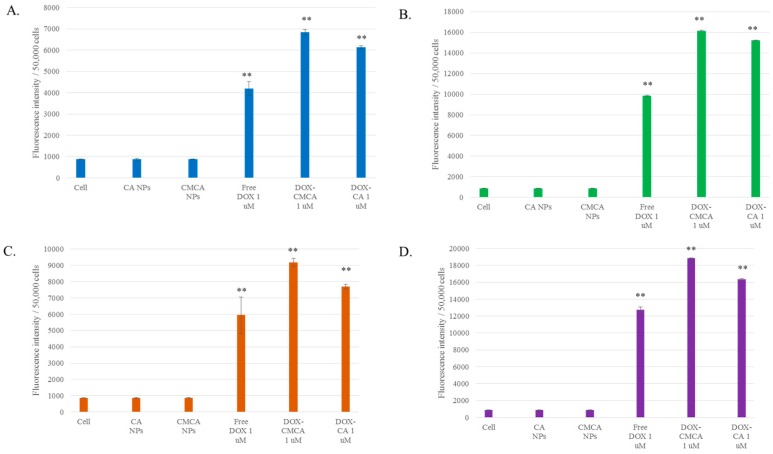
Cellular uptake of free DOX, DOX-CA NPs and DOX-CMCA NPs. (**A**) fluorescence intensity measuring DOX (1 µM) uptake with MCF-7 cells at 1 h of treatment; (**B**) fluorescence intensity measuring DOX (5 µM) uptake with MCF-7 cells at 1 h of treatment; (**C**) fluorescence intensity measuring DOX (1 µM) uptake with MCF-7 cells at 4 h of treatment; (**D**) fluorescence intensity measuring DOX (5 µM) uptake with MCF-7 cells at 4 h of treatment. Values were significant (*) at *p*-value < 0.05, extremely significant (**) at *p*-value < 0.0001 vs. untreated cells at CI of 95%.

The cytotoxic effects of free DOX, DOX-loaded CA, DOX-loaded CMCA and DOX-loaded SMCA are shown in Figures 6–8, with cell viability for CA, CMCA and SMCA NPs found to be 86%, 88% and 92%, respectively, in the MCF-7 cell line (Figure 5A, Figure 6A and Figure 7A). The results showed that the toxicity of DOX-loaded CMCA NPs was much higher than that of DOX-loaded CA, DOX-loaded SMCA and free DOX. Free DOX caused 6% and 5% cytotoxicity at 1 pM concentration and 60% and 50% cytotoxicity at 1 µM concentration in 4T1 and MCF-7 cells, respectively. The same concentrations of DOX in CMCA-loaded forms resulted in 50% (CI 95%, *p* < 0.0001 vs. same treatment of free drug) and 48% (CI 95%, *p* < 0.0001 vs. same treatment of free drug) cytotoxicity at 1 nM concentration and 85% (CI 95%, *p* < 0.0001 vs. same treatment of free drug) and 88% (CI 95%, *p* < 0.0001 vs. same treatment of free drug) cytotoxicity at 1 µM concentration in 4T1 and MCF-7 cells, respectively. DOX-loaded CA NPs showed almost 76% (CI 95%, *p* < 0.0001 vs. same treatment of free drug) cytotoxicity in 4T1 and MCF-7 cell line at 1 µM concentration, which is comparatively 9% lower cytotoxicity than DOX-loaded CMCA NPs (Figure 6 and Figure 7). The DOX-incorporated SMCA NPs exhibited almost 44% (CI 95%, *p* < 0.01 vs. same treatment of free drug) and 50% (CI 95%, *p* < 0.001 vs. same treatment of free drug) cytotoxicity at 10 nM concentration in MCF-7 cell and 4T1 cells, respectively (Figure 7).

The enhancement of % cytotoxicity for free DOX and DOX-loaded CA, CMCA and SMCA NPs in two different cell lines has been summarized in Table 1, Table 2 and Table 3. DOX-incorporated CA NPs and DOX-incorporated CMCA NPs demonstrated the concentration-independent enhancement in cytotoxicity in both cell lines, whereas DOX-loaded SMCA NPs revealed concentration-dependent enhanced cytotoxicity. Enhanced cytotoxicity was reported for every drug concentration for DOX-loaded apatite complexes compared to the free drug. The most significant enhancement in cytotoxicity was 11% (CI 95%, *p <* 0.0001 vs. same treatment of free drug) and 25% (CI 95%, *p <* 0.0001 vs. same treatment of free drug) noticed with 1 µM concentration of DOX-loaded into CA and CMCA NPs, respectively, compared to the free drug in MCF-7 cell line. DOX-loaded SMCA NPs demonstrated 17% (CI 95%, *p <* 0.0001 vs. same treatment of free drug) at 10 pM concentration and 22% (CI 95%, *p <* 0.0001 vs. same treatment of free drug) enhanced cytotoxicity at 1 µM concentration of DOX in MCF-7 cells and 4T1 cells, respectively. This enhancement in cytotoxicity could be as a result of the endocytosis-facilitated cellular uptake of DOX [12,13].

Different concentrations of DOX (1 pM–1 µM) were used for evaluating the therapeutic potency of free and NPs-bound forms of the drug in MCF-7 cells. A dose-dependent cytotoxicity was observed with all the NPs after 48 h of treatment. In MCF-7 cell line, IC_50_ for DOX was achieved at 1 µM, causing almost 50% toxicity compared to the untreated control. DOX-loaded CA and SMCA NPs caused approximately 50% cell death at 10 nM concentration of DOX, whereas DOX-loaded CMCA caused 50% cell death at 1 nM concentration of the drug compared to the untreated control. Based on this result, CMCA NPs prepared in the presence of 1 nM DOX was found to confer equivalent cytotoxicity of 1 µM of free DOX (Figure 5, Figure 6 and Figure 7).

### 3.5. Cellular Uptake Study by Quantitative Fluorescence Intensity Measurement of DOX

MCF-7 cells were treated with 1 µM and 5 µM concentrations of DOX, DOX-loaded CA and DOX-loaded CMCA NPs to assess cellular uptake efficacy of the drug-loaded NPs in comparison to the free drug. As shown in Figure 8, Table 4 and Table 5, higher uptake of DOX-loaded CMCA NPs was noticed compared to free DOX and DOX-loaded CA. The cellular uptake of free DOX was 22% (CI 95%, *p <* 0.0001 vs. untreated control) and 31% (CI 95%, *p <* 0.0001 vs. untreated control) for 1 µM concentration, and 24% (CI 95%, *p <* 0.0001 vs. untreated control) and 32% (CI 95%, *p <* 0.0001 vs. untreated control) for 5 µM concentration at 1 h and 4 h of treatments, respectively. CMCA-facilitated cellular uptake for DOX was 36% (CI 95%, *p <* 0.0001 vs. untreated control) and 48% (CI 95%, *p <* 0.0001 vs. untreated control) for 1 µM concentration and 40% (CI 95%, *p <* 0.0001 vs. untreated control), and 47% (CI 95%, *p <* 0.0001 vs. untreated control) for 5 µM concentration at 1 h and 4 h of treatments, respectively. CMCA at first hour of treatment showed 4% and 14% more cellular uptake for 1 µM DOX than DOX-loaded CA and free DOX, respectively. The cells treated with DOX-free NPs displayed similar fluorescence intensity as the untreated control cells.

### 3.6. Cellular Uptake Study by Fluorescence Microscopy

**Figure 9:**
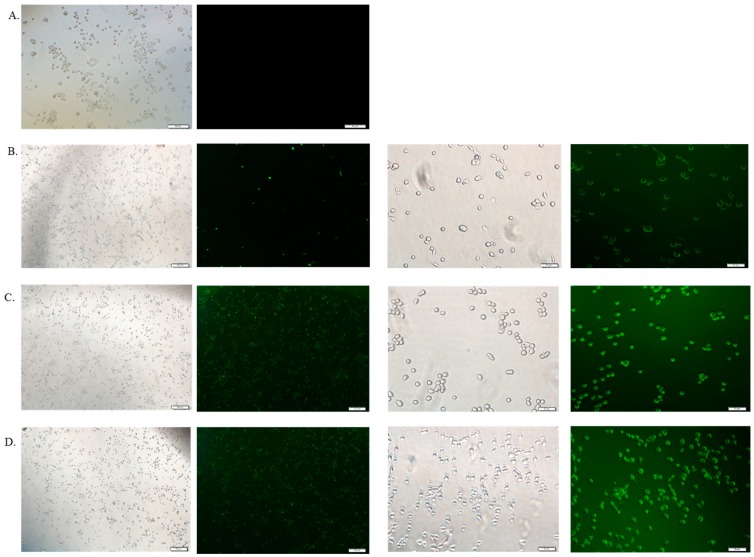
Fluorescence image analysis of MCF-7 cells incubated with media only (**A**), DOX 1 µM (**B**), DOX-loaded CA NPs (1 µM) (**C**) and DOX-loaded CMCA NPs (1µM) (**D**) at 37 °C for 1 h with 4× and 20× magnifications at a scale bar of 100 µm and 20 µm.

**Figure 10:**
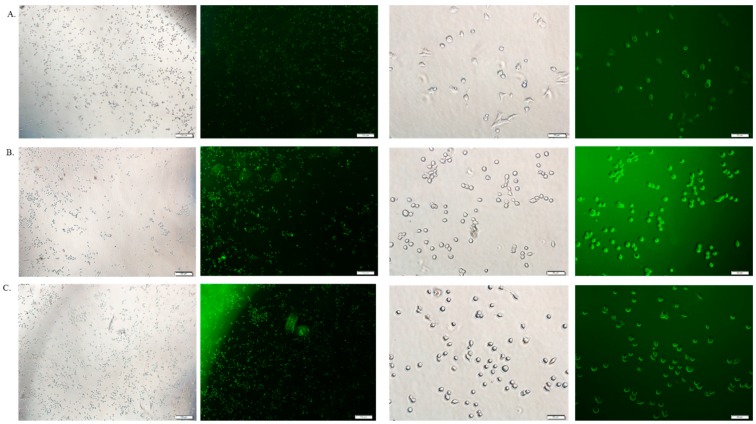
Fluorescence image analysis of MCF-7 cells incubated with DOX 5 µM (**A**), DOX-loaded CA NPs (5 µM); (**B**) and DOX-loaded CMCA NPs (5 µM); (**C**) at 37 °C for 1 h with 4× and 20× magnifications at a scale bar of 100 µm and 20 µm.

**Figure 11:**
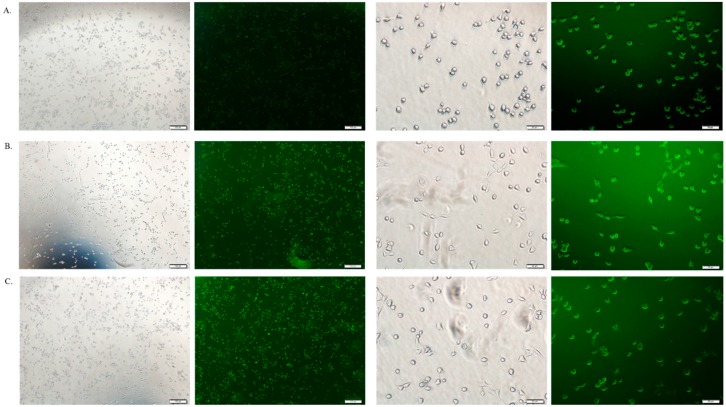
Fluorescence image analysis of MCF-7 cells incubated with DOX 1 µM (**A**), DOX-loaded CA NPs (1 µM); (**B**) and DOX-loaded CMCA NPs (1 µM); (**C**) at 37 °C for 4 h with 4× and 20× magnifications at a scale bar of 100 µm and 20 µm.

**Figure 12:**
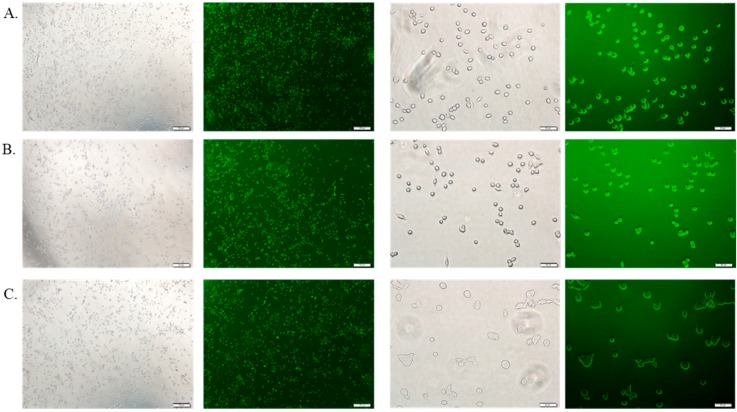
Fluorescence image analysis of MCF-7 cells incubated with DOX 5 µM (**A**), DOX-loaded CA NPs (5 µM); (**B**) and DOX-loaded CMCA NPs (5 µM); (**C**) at 37 °C for 4 h with 4× and 20× magnifications at a scale bar of 100 µm and 20 µm.

The cellular uptake of therapeutics depends on the amount of drug binding to the particles and the size of the drug-bound particle. Free DOX, DOX-loaded CA and DOX-loaded CMCA NPs were treated with MCF-7 cells to investigate the drug internalization by observing cellular fluorescence. As shown in Figure 9, Figure 10, Figure 11 and Figure 12, the amount of free DOX internalization was time-dependent. The uptake of free DOX in the cytoplasm is comparatively high at 4 h of treatment than at 1 h of treatment. Fluorescence images of DOX-apatite complexes displayed enhanced cellular uptake at 1 h treatment in comparison to free drug (Figure 9). The stronger fluorescence intensity of drug molecules within cells was observed after 4 h of incubation (Figure 11 and Figure 12), indicating that DOX-apatite complexes could be efficiently taken up by tumor cells. The cells that were kept untreated and treated with NPs did not show any fluorescence.

### 3.7. Estimation of Unbound Citrate in CMCA NPs

The standard curve was made using peak areas of different concentrations of sodium citrate in HPLC analysis. HPLC analysis was performed using the above-mentioned protocol to detect any interference in the peak area by only DMEM media, CA NPs. No peak was detected for DMEM media and CA NPs. At 8 mM and 16 mM concentrations of sodium citrate, no peak was found for citrate in the supernatant (data not shown), indicating that all sodium citrate that has a strong affinity for Ca^2+^ due to possession of 3 carboxylic acids, was bound to the NPs [23].

### 3.8. Confirmation of pH-Sensitivity of CA, CMCA and SMCA NPs

**Figure 13:**
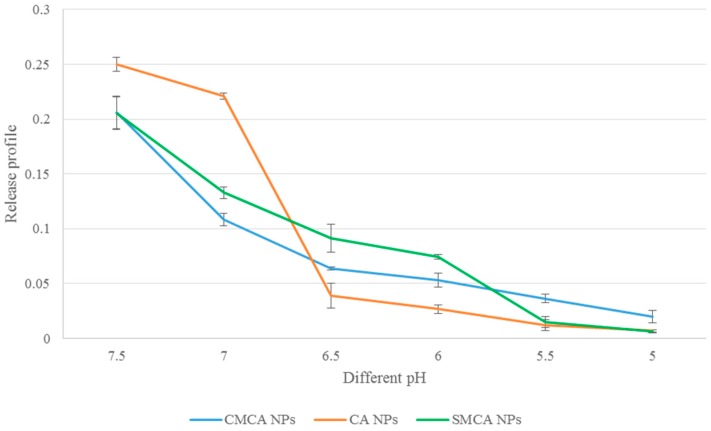
pH-dependent release of CA, CMCA and SMCA NPs.

CA, CMCA and SMCA NPs were prepared and subjected to different pHs to confirm their pH sensitivity. The turbidity test showed that the nanoparticles were stable at blood (physiological) pH 7.4, but dissolved at endosomal acidic pH (6.5–5.0) (Figure 13). The results confirmed that the CMCA and SMCA NPs were rapidly dissolved in the weak acidic environment. Moreover, CA, CMCA and SMCA NPs exhibited almost 84.4%, 69.41% and 55.56% release in pH 6.5, respectively, and 97.2%, 90.29% and 96.93% release in pH 5 within 5 min, respectively. It is clear that carbonate apatite-based NPs possess the unique characteristic of pH sensitivity that in turn helps the NPs to release the loaded-drug into the cytoplasm.

### 3.9. pH-Dependent DOX Release from NPs

**Figure 14:**
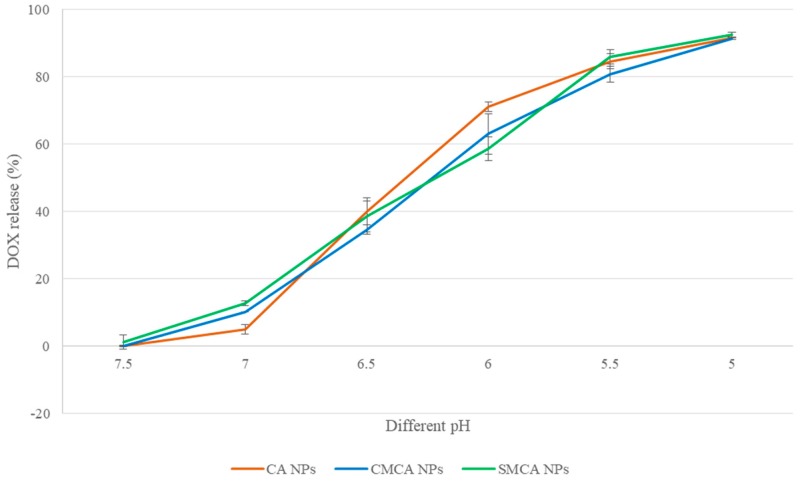
pH-dependent DOX-release from CA, CMCA and SMCA NPs.

At pH 7.4, only 0.15%, 0.05% and 1.23% of DOX were released from CA, CMCA and SMCA NPs, respectively. The low release rate of DOX is beneficial for intravenous treatment because the physiological pH of blood and healthy tissues is pH 7.4. By contrast, 40.18%, 34.75% and 38.61% of total DOX was released from CA, CMCA and SMCA NPs, respectively, at pH 6.5 because of the significant particle dissoluion at this particular pH, as shown in Figure 14. Almost 92% of total DOX was released from all the NPs at pH 5.0. The pH-dependent DOX release from the apatite-based NPs potentially facilitated accumulation of active (free) drug inside the tumor cells and reduced the toxicity to normal cells.

### 3.10. Characterization of SMCA NPs by FTIR

**Figure 15:**
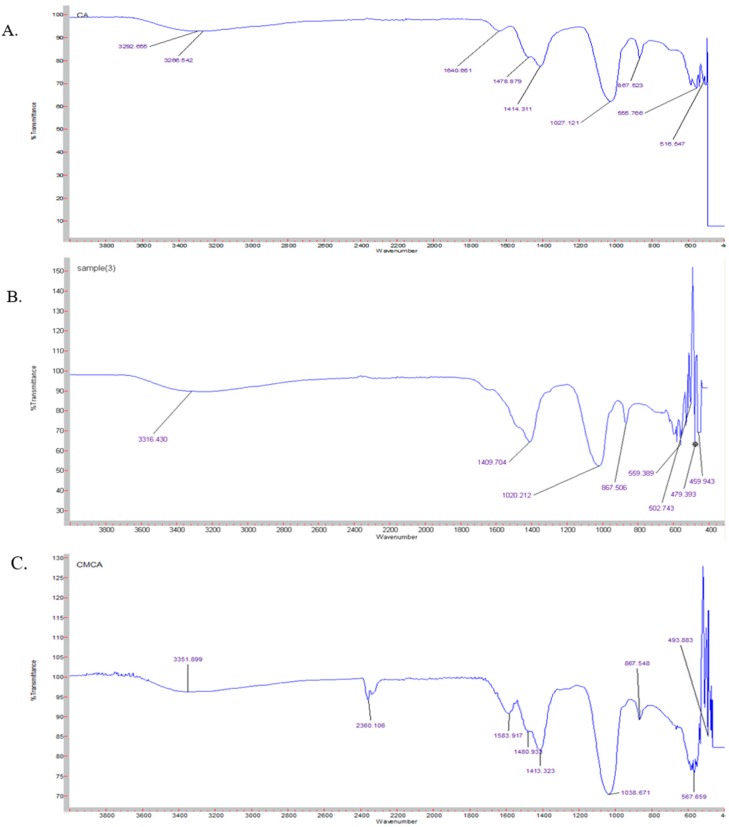
The FTIR spectrum. (**A**) the FTIR spectrum of CA NPs; (**B**) the FTIR spectrum of SMCA NPs; (**C**) the FTIR spectrum of CMCA NPs.

To confirm the association of citrate and succinate with CA NPs, we have carried FTIR of CA, CMCA and SMCA NPs (Figure 15) along with sodium succinate and sodium citrate (data not shown). The formation of CA NPs was identified by the absorption peaks at 1640, 1478 and 1414 cm^−1^, representing the presence of carbonate (–CO^3−^) in the apatite particles (Figure 15A). However, the absorbance peaks of CA NPs at 1640 and 1478 cm^−1^ disappeared and the absorbance peak at 1414 cm^−1^ was shifted to 1409 cm^−1^ for SMCA NPs, suggesting the masking effect of succinate with the carbonate group of apatite particles (Figure 15B). CA NPs obtained the absorbance peak at 3292 and 3266 cm^−1^ due to the transmittance of OH stretching which has widened after sodium succinate reacted with CA NPs to form SMCA NPs. In case of CMCA NPs, the absorbance peaks of CA NPs at 1640 and 1027 cm^−1^ were shifted to 1583 and 1038 cm^−1^ respectively for CMCA NPs, indicating the binding of citrate with the apatite particles (Figure 15C). CA, CMCA and SMCA NPs showed the common absorbance peak at 867 cm^−1^, signifying the presence of a phosphate group.

### 3.11. Characterization of CA, CMCA and SMCA NPs by FE-SEM

**Figure 16:**
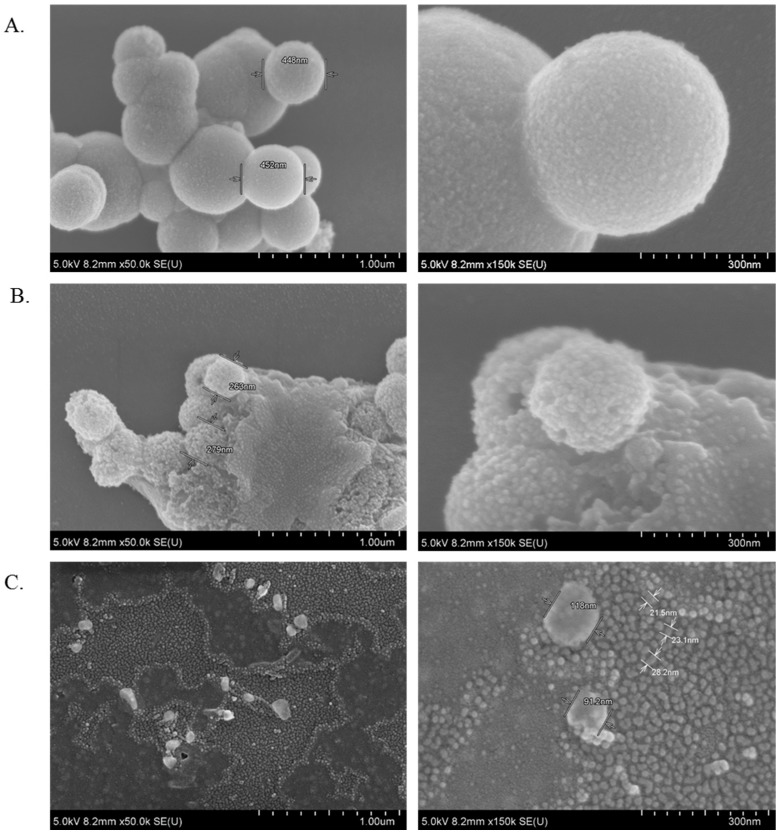
FE-SEM (Field emission scanning electron microscopy) micrographs of NPs. (**A**) micrographs of CA NPs at 1 µm and 300 nm scale; (**B**) micrographs of SMCA NPs at 1 µm and 300 nm scale; (**C**) micrographs CMCA of NPsat 1 µm and 300 nm scale.

FE-SEM images were taken for the characterization of the morphology, chemical nature and size of CA, CMCA and SMCA NPs (Figure 16). As shown in Figure 16A, CA NPs were very uniform and spherical in shape with the average particle diameter of 448–452 nm. The size of spherical SMCA NPs was around 263–279 nm, offering a larger surface area for more drug binding compared to the former (Figure 16B). CMCA NPs were formed with the smallest diameter ranging from 23 to 118 nm, offering the highest surface area from numerous tiny particles (Figure 16C) and thereby more drug binding opportunity [27]. Thus, it could be concluded that CMCA NPs are apparently the most suitable apatite-based NPs in terms of morphology, particle size and particle numbers.

### 3.12. Characterization of CA, CMCA and SMCA NPs by DLS

**Figure 17:**
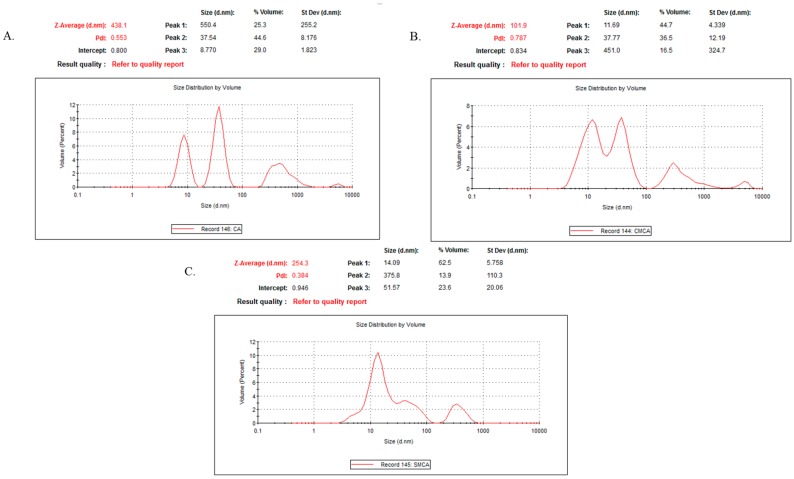
Particle size distributions determined by DLS (Dynamic light scattering). (**A**) DLS curve of CA NPs; (**B**) DLS curve of CMCA NPs. (**C**) DLS curve of SMCA NPs.

The light intensity scattered by the sample particles is used to analyze particle size distributions (PSD). However, DLS measurement is more sensitive to larger particles. As such, overestimation of particle size might happen sometimes especially for inorganic NPs [28,29]. The polydispersity index (PdI) values for CA, CMCA and SMCA NPs were 0.553, 0.787 and 0.384, respectively (Figure 17), suggesting the polydisperse nature of the nanoformulations. The PdI value of CA NPs indicates agglomeration of the particles as the polydisperse system had a tendency to aggregate more than a monodisperse system (Figure 17A), which could be supported by SEM micrographs of CA NPs (Figure 16A), whereas the PdI value of CMCA NPs could be justified by formation of dense particles in different sizes ranging from 23 to 107 nm without particle agglomeration (Figure 17B). The PdI value of SMCA NPs was less than that of either CA NPs or CMCA NPs, due to the uniformity in particle size (Figure 17C).

A correlation was observed between the *z*-average size and the SEM image of each of the particle type, suggesting that there was no overestimation in particle size analysis for CA, CMCA and SMCA NPs.

## 4. Discussion

Inorganic CA NPs have emerged as an attractive drug delivery system due to their biocompatibility, pH-sensitivity and a wide range of binding affinity towards drugs through electrostatic interactions. In addition, apatite-based inorganic NPs have a unique property of releasing drugs at the acidic environment of endosomes after cellular internalization [18].

Different parameters were optimized in earlier studies to improve the growth kinetics of CA NPs such as salt concentration, pH of the media, incubation time and temperature, and replacement of Ca^2+^, a divalent cation, with another divalent cation such as magnesium (Mg^2+^) [30] or strontium (Sr^2+^) [20] to form smaller NPs. However, a recent study on intracellular transport of Anastrozole and Gemcitabine into breast cancer cells using CA NPs [13] has shown limited enhancement in drug-induced cytotoxicity, particularly owing to the lack of proper control on growth kinetics of the particles, motivating us to further improve the current technology.

DOX-loaded CMCA and SMCA NPs were formed in a bicarbonate-buffered medium at fixed concentrations of inorganic phosphate (PO_4_^3−^), bicarbonate (HCO_3_^−^) and Ca^2+^, along with varying concentrations of citrate (C_6_H_5_O_7_^3−^) and succinate (C_4_H_6_O_4_) ions, through incubation at 37 °C. The turbidity analysis was conducted to ensure the formulation of the different particles and the possible drug binding with CA, CMCA and SMCA NPs (Figure 1A). The gradual increase in turbidity of DOX-particle complex with increasing drug concentrations is indicative of drug–particle interactions, which would need to be further supported by the size and zeta potential of DOX-incorporated NPs, and DOX binding affinity for the NPs. Drug binding with NPs depends on a few parameters such as drug solubility in the liquid phase, drug–particle interaction and the presence of functional group(s) [31]. DOX could bind with generating CA, CMCA and SMCA NPs through ionic interactions of its protonated amine groups with the anionic (PO_4_^3−^/HCO_3_^−^-rich) domains of the NPs, further inducing the particle formulation. In order to ensure DOX binding with apatite-based NPs, the amount of bound drug was determined by sedimenting the drug-particle complex and detecting the fluorescence intensity by dissolving the pellet with EDTA in PBS (Figure 4). Flurospectrophotometric analysis showed significant dose-dependent binding affinity of DOX to CMCA and SMCA NPs compared to CA NPs, with CMCA nanoparticles possessing higher affinity than SMCA NPs for the drugs, accounting for a dose-dependent decrease in particle size as well as electropositivity in surface charge of the drug-loaded NPs. 

NPs are directly administered as nanocarriers into the systemic circulation to release the payload into the tumor tissue to achieve enhanced therapeutic efficacy by increasing circulation half-life of the drug while reducing its unwanted accumulation in healthy tissues [17,32,33]. Although NPs are unable to cross the tight junction between the endothelial cells of healthy vascular lining due to their comparative larger size, they can easily go through the leaky vasculature tumor region through EPR effect and accumulate in the tumor tissue for prolonged period of time due to the absence or dysfunction of the lymphatic system inside a tumor [33]. This multi-step process depends on the physical properties of nanocarriers such as size, shape and surface charge of the NPs [34,35]. Particle size within the range of 20 nm to 1 µM is crucial for prolonging circulation time, circumventing opsonization and reducing the uptake rate of MPS [34,36,37]. DOX-loaded CMCA NPs, in particular, were found to be within a 30–100 nm range (Figure 3C) and, therefore, would be a potent candidate for further evaluation in animal models. The surface charges for DOX-incorporated CA, CMCA NPs were more electropositive between −5 to −9 mV in our current study (Figure 3B,D), which could be due to the ionic bonding in between protonated amine group of DOX and the negatively charged domain (PO_4_^3−^, CO_3_^2−^) of apatite-based NPs.

For conferring significant cytotoxicity to cancer cells, while reducing side effects to healthy tissues and overcoming the multidrug resistance, it is a prerequisite for the apatite-based nanocarriers to release DOX into the targeted cells through the pH-responsive dissolution of the NPs [38,39,40,41]. In order to confirm pH sensitivity of the NPs at the acidic environment, pH-dependent particle dissolution profiles were determined by turbidity analysis. Under normal physiological conditions, CA, CMCA and SMCA NPs are stable, but at acidic pH of the intracellular microenvironments (endosomes), the phosphate and bicarbonate ions present in the apatite structure tend to accept excess H^+^ ion, causing the particles to be dissolved [42]. Moreover, the cation (Ca^2+^) and anions (PO_4_^3−^, CO_3_^2−^) released from the particles might create osmotic pressure across the endosomal membrane, which, in turn, could lead to end some swelling and rupture, releasing the payloads into the cytoplasm [43,44]. However, after the dissolution of the NPs in the endosome, DOX might also enter into the cytoplasm through passive diffusion through the endosomal membrane.

Several studies were conducted for targeted delivery of DOX by encapsulating into different nanocarriers to reduce its biodistribution in normal healthy tissues. Recently, halloysite nanotubes (HNTs) received much attention due to their larger surface area, non-toxicity and good thermal stability. Apart from this, HNTs can easily encapsulate drugs and can sustain the drug release by preventing rapid enzymatic degradation in vivo [45,46]. Li et al. [46] modified HNTs with soybean phospholipid to generate HNTs/DOX/LIP complexes and noticed almost 22.01% ± 0.43 DOX loading efficiency for HNTs and HNTs/DOX/LIP. However, HNTs negatively charged surface (−16.5 ± 1.2 mV) requires positively charged drugs to bind, restricting the binding opportunity for negatively charged drugs. In another study, HNTs were conjugated with poly(ethylene glycol) (PEG) and folate (FA) to formulate HNTs-PEG-FA complexes for targeted DOX delivery into breast tumors [47]. HNTs-PEG-FA complexes possessed only 3% DOX loading efficiency and almost 35% cell viability for 8.0 × 10^3^ MCF-7 cells/well at a concentration of 10 µg/mL DOX. Organic-inorganic hybrid nanocarriers such as mesoporous silica nanoparticles self-assembled with stearic acid-g-chitosan (CS-SA) to compose CS-SA/SiO_2_ NPs, were used to carry DOX into tumor sites [48]. The DOX loading amount of CS-SA/SiO_2_ was nearly 4.5%, with IC_50_ values achieved by using around 1 µg/mL concentration of DOX against A549 cells. Comparing to these studies, apatite-based NPs showed promising results in terms of drug binding, surface charge and cytotoxicity. CA, SMCA and CMCA NPs revealed almost 26.25%, 28.20% and 31.38% binding affinity for DOX, respectively, and more electropositive surface charge between −5 to −9 mV for DOX-loaded NPs. Higher cytotoxicity was observed for CMCA NPs using a very low concentration of DOX. Almost 51% and 12% of cell viability for 5.0 × 10^3^ MCF-7 cells seeded per well were achieved at drug concentrations of 1 nM (0.6 ng/mL) and 1 µM (0.6 µg/mL), respectively.

DOX-loaded CMCA and CA NPs revealed time-dependent and dose-dependent cellular uptake in MCF 7 cells. Interestingly, at 4 h of the treatment, DOX-loaded CMCA NPs achieved almost 50% cellular uptake (Table 5, Figure 8C,D). Fluorescence intensity analysis was performed to quantitate time-dependent cellular uptake of free DOX, DOX-loaded CA NPs and DOX-loaded CMCA NPs in MCF-7 cells at different concentrations of the drug. As shown in Table 4 and Table 5, free DOX demonstrated cellular uptake efficiency of almost 22.4% (CI 95%, *p <* 0.0001 vs. untreated control) for 1 µM and 24% (CI 95%, *p <* 0.0001 vs. untreated control) for 5 µM concentration at 1 h incubation, which gradually decreased with a further increase in the incubation time. On the contrary, the fluorescence intensity for both DOX-loaded CMCA and CA NPs was greatly enhanced with increasing treatment time in MCF-7 cells. Interestingly, although DOX-loaded CMCA showed the similar fluorescence intensity as DOX-loaded CA at 1 h time point with the different concentrations of the drug, at 4 h incubation, DOX-loaded CMCA NPs showed almost 7% more cellular uptake than DOX-loaded CA NPs for 1 µM and 5 µM concentration. The results indicated that DOX-loaded CMCA NPs were more efficiently taken up by the cells via endocytosis than DOX-loaded CA NPs [49,50,51]. Apart from this, enhancement in cytotoxicity revealed 25% more cytotoxicity with DOX-bound CMCA NPs than the same amount of free drug, which could be explained by the endocytosis-facilitated cellular uptake of DOX, a process considered to be more efficient than the concentration-dependent passive diffusion of a particular drug [18,19]. This enhanced cytotoxicity could result from the ability of small size CMCA NPs to adsorb the drug in sufficient amounts and subsequently undergo effective cellular internalization via endocytosis [18]. The release of DOX from CA, CMCA and SMCA NPs were likely due to their dissolution at endosomal acidic pH [52,53,54], since the particles were found to follow the similar trend of rapid degradation with a small decrease in pH (Figure 13).

## 5. Conclusions

We have successfully developed a pH-sensitive, nano-sized drug carrier based on CMCA that showed significant drug loading efficiency, enhanced cellular uptake and rapid dissolution at endosomal acidic pH to effectively release the drug, resulting in efficient cytotoxicity comparing to original CA as well as SMCA NPs. Thus, CMCA is a highly promising pH-sensitive nanoparticle for anti-cancer drug delivery and hence demands further investigation in preclinical cancer models.

## Figures and Tables

**Table 1:** Enhancement of cytotoxicity (%) for DOX (Doxorubicin)-loaded CMCA (Citrate-modified carbonate apatite) NPs (nanoparticles).

Concentration of DOX	MCF-7	4T1
1 pM	13.35 ± 1.92	4.14 ± 1.4
10 pM	16.90 ± 1.34	12.42 ± 1.62
100 pM	10.85 ± 1.87	13.45 ± 2.27
1 nM	15.66 ± 2.69	17.72 ± 2.58
10 nM	15.12 ± 3.25	15.78 ± 4.32
100 nM	20.99 ± 1.93	19.40 ±1.34
1 µM	25.62 ± 0.82	11.77 ± 1.25

MCF-7: Michigan Cancer Foundation-7 (human breast cancer cell line).

**Table 2:** Enhancement of cytotoxicity (%) for DOX-loaded CA (Carbonate apatite) NPs.

Concentration of DOX	MCF-7	4T1
1 pM	1.78 ± 2.52	0.91 ± 2.58
10 pM	2.67 ± 3.5	7.37 ± 0.22
100 pM	1.60 ± 2.97	3.62 ± 3.01
1 nM	1.42 ± 1.92	1.16 ± 1.24
10 nM	1.94 ± 2.7	3.23 ±2.20
100 nM	1.11 ± 2.93	3.62 ± 4.25
1 µM	11.03 ± 1.34	5.04 ± 5.1

**Table 3:** Enhancement of cytotoxicity (%) for DOX-loaded SMCA (Succinate-modified carbonate apatite) NPs.

Concentration of DOX	MCF-7	4T1
1 pM	2.89 ± 6.68	2.99 ± 6.76
10 pM	17.33 ± 2.24	7.49 ± 2.79
100 pM	7.64 ± 2.93	10.64 ± 1.04
1 nM	7.52 ± 0.54	11.64 ± 1.94
10 nM	6.25 ± 2.56	16.93 ± 1.41
100 nM	8.55 ± 2.11	20.88 ± 7.48
1 µM	7.60 ± 2.92	22.53 ± 1.29

**Table 4:** Cellular uptake for DOX 1 µM, DOX (1 µM)–CA NPs and DOX (1 µM)–CMCA NPs after 1 h and 4 h of treatment.

Formulation	% Cellular Uptake 1 h of Treatment	% Cellular Uptake 4 h of Treatment
DOX	22.4 ± 1.73	31.71 ± 5.92
DOX-CA	32.69 ± 0.38	41.09 ± 0.79
DOX-CMCA	36.45 ± 0.72	48.93 ± 1.32

**Table 5:** Cellular uptake for DOX 5 µM, DOX (5 µM)–CA NPs and DOX (5 µM)–CMCA NPs after 1 h and 4 h of treatment.

Formulation	% Cellular Uptake 1 h of Treatment	% Cellular Uptake 4 h of Treatment
DOX	24.75 ± 0.14	32.07 ± 0.82
DOX-CA	38.26 ± 0.07	41.14 ± 0.26
DOX-CMCA	40.64 ± 0.12	47.41 ± 0.11
